# Real-World Treatment Patterns and Physician Preferences for Biologics in Moderate-to-Severe Inflammatory Bowel Disease: Retrospective Chart Review in Europe

**DOI:** 10.1093/crocol/otac001

**Published:** 2022-01-27

**Authors:** Lynn Huynh, Steve Hass, Laurent Peyrin-Biroulet, Mei Sheng Duh, Heather Sipsma, Mu Cheng, Angie Lax, Arpita Nag

**Affiliations:** 1 Analysis Group, Inc., Boston, Massachusetts, USA; 2 H. E. Outcomes, LLC, Los Angeles, California, USA; 3 Department of Gastroenterology, Nancy University Hospital, Université de Lorraine, Nancy, France; 4 Takeda Development Center Americas, Lexington, Massachusetts, USA

**Keywords:** biological therapy, biosimilar pharmaceuticals, ulcerative colitis, Crohn’s disease, physicians’ practice patterns

## Abstract

**Background:**

With many options available for treating inflammatory bowel disease (IBD) in Europe, this study sought to characterize physician treatment preferences and real-world treatment patterns in patients with moderate-to-severe ulcerative colitis (UC) and Crohn’s disease (CD).

**Methods:**

This was a retrospective, noninterventional, physician-administered study. Gastroenterologists and general practitioners (*n* = 348) in France, Germany, and the United Kingdom provided information on treatment preferences and extracted information from records of patients with moderate-to-severe UC (*n* = 587) or CD (*n* = 417) who had received biologic, biosimilar or Janus kinase inhibitor therapies (2014–2019) and had IBD-related medical history available 6 months before and after treatment initiation.

**Results:**

Physicians largely preferred infliximab and adalimumab or their biosimilars as first-line therapy for UC (originators, 65.8%; biosimilars, 26.1%) and CD (originators, 61.8%; biosimilars, 30.5%). Effectiveness was the most cited reason for treatment preference (92%–93% of physicians). Three-quarters of patients (UC, 75.8%; CD, 73.6%) received infliximab or adalimumab originators in the first line, with more patients receiving infliximab biosimilars than adalimumab biosimilars (12.4%–12.5% and 0.5%–4.1%, respectively, across UC and CD). Persistence was longer for first-line infliximab than adalimumab (UC, 26.6 vs 21.2 months; CD, 31.2 vs 26.7 months) and was generally shorter for their respective biosimilars. Nonbiologic treatments were used in combination with biologics in 14.1% (UC) and 11.5% (CD) of patients. Most patients received 1 biologic therapy (UC, 90.6%; CD, 83.2%); only 9.4% (UC) and 16.8% (CD) received a second biologic.

**Conclusions:**

Infliximab and adalimumab originators dominated first-line biologic therapy for moderate-to-severe UC and CD. Understanding real-world treatment patterns can help assess new treatment uptake and suggest opportunities for improving treatment.

## Introduction

Inflammatory bowel disease (IBD) is a chronic and disabling inflammatory disorder encompassing ulcerative colitis (UC) and Crohn’s disease (CD).^1,2^ Guidelines are generally consistent in their recommendations for the treatment of IBD,^3–6^ which are summarized below. Glucocorticoids such as prednisolone and budesonide are used in the first instance for both moderate-to-severe UC and CD, although long-term use is not recommended owing to toxicity and dependence. Patients with UC can be maintained with 5-aminosalicylic acid (most commonly mesalazine). Patients with CD are maintained with thiopurines (azathioprine or mercaptopurine), and methotrexate may be considered to minimize the risk of flare during withdrawal of prednisolone. Patients with UC maintained with high-dose mesalazine and requiring 2 or more courses of corticosteroids as a bridging treatment in the past year (steroid excess is considered to be 2 or more courses of steroid over 1 year) are escalated most commonly to antitumor necrosis factor (TNF) biologics (eg, infliximab, adalimumab, and golimumab). These are then followed by a different anti-TNF treatment, anti-integrin therapy (vedolizumab), or a Janus kinase (JAK) inhibitor (tofacitinib) as second-line biologic treatment (note that ustekinumab was granted an expanded use for the treatment of moderate-to-severe active UC by the European Commission in September 2019, and was approved by the US Food and Drug Administration in October 2019; as such, the broadened use of ustekinumab was not evaluated within the current study time period). Similarly, patients with CD who have not responded to conventional therapy, and those with extensive disease and poor prognosis, are generally considered early for first-line anti-TNF treatment, anti-interleukin-12/23 (ustekinumab), or vedolizumab, with switching to ustekinumab or vedolizumab being recommended upon failure of anti-TNF therapy. When switching biologic treatment for both UC and CD, the choice should be made based on predicted effectiveness, tolerability, cost, and patient preference.

Although clinically effective, biologic treatments are costly, and access in some countries can be limited owing to budgetary constraints.^7^ In recent years, several biosimilar drugs have been developed and approved for use in Europe for the treatment of IBD.^8^ As a result, a range of less costly treatment options is now available for the management of IBD, which will likely enable more widespread use of anti-TNF biologics. However, available real-world evidence suggests that originator biologics continue to be considered the standard of care.^7,9,10^ The CHart review Evaluating Real-world Use of Biologics in moderate-to-severe IBD (CHERUB-IBD) study aimed to generate real-world evidence to characterize physician preferences for the management of moderate-to-severe UC and CD and to describe the treatment pathways and patterns of biologic use (including combination treatment, and frequency of and reasons for biologic treatment switches) in patients with moderate-to-severe UC and CD in France, Germany, and the United Kingdom.

## Materials and Methods

### Study Design

This was a retrospective, double-blinded (ie, physicians were blinded to the study sponsor information and the study sponsor was blinded to the physicians recruited to participate), noninterventional, physician-administered chart review conducted in France, Germany, and the United Kingdom. These 3 countries were selected based on a feasibility assessment (ie, a short questionnaire was administered to assess the potential number of patients with IBD in these countries). The assessment showed there to be a reasonable number of physicians who treated patients with IBD with originators and biosimilars in these countries; given the sizable target population, the decision was made to conduct the physician panel-based chart review study in France, Germany, and the United Kingdom.

Gastroenterologists and general practitioners (GPs) in France, Germany, and the United Kingdom were recruited through Medefield (New York, NY), a physician panel vendor, over a 7-week period in October and November 2019. Recruitment of gastroenterologists was prioritized (ie, gastroenterologists were approached first, followed by GPs) as gastrointestinal-related issues and treatments were more likely to be monitored and documented by gastroenterologists. Physician eligibility was confirmed via a screening survey; physicians were required to have treated at least 10 patients with a diagnosis of moderate-to-severe UC or CD, and to have prescribed at least 1 of the following biologic or JAK inhibitor treatments to their patients: adalimumab, adalimumab biosimilars, infliximab, infliximab biosimilars, golimumab, vedolizumab, tofacitinib, or ustekinumab. After establishing eligibility, physicians provided information regarding the number of patients they treated per year and their perceptions regarding treatment (including preferred first-line treatment for patients with moderate-to-severe UC or CD, primary reason for treatment preference and type of treatment most likely to be prescribed after failure of anti-TNF treatment). They then extracted information on demographic and clinical characteristics and treatment patterns from the charts of eligible patients.

### Study Population

For patient records to be considered eligible for inclusion in the study, patients were required to have moderate-to-severe UC (defined, if scores were available, as a total Mayo Endoscopic score ≥6, with an endoscopic subscore ≥2, a rectal bleeding subscore ≥1, and a stool frequency subscore ≥1) or CD (defined, if scores were available, as a Crohn’s Disease Activity Index [CDAI] total score of ≥220 to ≤450 or a Harvey–Bradshaw Index [HBI] score ≥8), to be aged ≥18 years at moderate-to-severe CD or UC diagnosis, and to have received one of the following biologic or JAK inhibitor treatments: adalimumab (or biosimilars), infliximab (or biosimilars), vedolizumab, ustekinumab (for CD only), or golimumab or tofacitinib (for UC only) from 2014 to 2019. Patients were also required to have a complete IBD-related medical history for 6 months before and after treatment initiation, be alive and be receiving treatment from the physician (defined as having a medical chart entry within the past 12 months). Patients with psoriasis, rheumatoid arthritis, psoriatic arthritis, polyarticular juvenile idiopathic arthritis or spondyloarthritis (including ankylosing spondylitis) were excluded. To minimize bias in the selection of patients, a computer-generated randomization scheme was employed. A web-based electronic case report form prompted physicians to select patient charts according to a randomized sequence of letters based on the patient’s last name. Physicians were instructed to select the first eligible patient whose last name began with the randomly generated letter and who met all eligibility criteria. If no such patient was available, they were instructed to move to the next letter in the sequence.

Physician participants provided patient data for the baseline period, defined as the 6 months before the initiation of the first biologic treatment for IBD (index date), and for the observation period, defined as the period of time from the index date to the last entry in the medical chart. Data were collected using an electronic case report form, which was translated and programmed into French and German to facilitate data collection in France and Germany, respectively. Demographics, IBD-related clinical characteristics, and information relating to nonbiologic treatments received were collected during the baseline period. Data collected during the observation period included information on up to 3 lines of biologic treatment, as well as the use of nonbiologic treatments. Specifically, data relating to the following treatment patterns were collected during the chart review: use of combination treatment (defined as the frequency of administration of more than 1 treatment at the same time, eg, immunomodulator plus biologic agent), treatment augmentation (the frequency of new treatments added to the index treatment), and treatment persistence and switching. Persistence was defined as the time to treatment discontinuation or switching and was reported for the first 2 lines of biologic treatment. For patients who did not discontinue or switch to a new biologic treatment, persistence was defined as time to death, last contact, or chart abstraction, whichever occurred first (ie, the end of the observation period). Frequency and reasons for switching were collected but were analyzed only in patients who initiated biologic treatment during or before 2017 to ensure sufficient follow-up time.

### Statistical Analyses

All physicians and patients meeting the eligibility criteria were included in the analyses. All statistical analyses were performed using descriptive statistics (including frequencies and percentages for categorical variables and means and SDs for continuous variables), with data analyzed separately for UC and CD, as well as by index treatment and physician type. Chi-square tests were conducted to evaluate differences in first treatment preference between gastroenterologists and GPs. Variables with unknown values were coded as missing/unknown. The number and proportion of missing/unknown values for each variable were then summarized.

### Ethical Considerations

The study was determined to be exempt from review by the New England Independent Review Board and complied with General Data Protection Regulation.

## Results

### Physician Characteristics

In total, 348 physicians (267 gastroenterologists [76.7%]; 81 GPs [23.3%]) were included in the study, with 33.0% (*n* = 115), 41.1% (*n* = 143), and 25.9% (*n* = 90) being from France, Germany, and the United Kingdom, respectively. The majority of physicians (87.9%) were practicing in a public setting. More than three-quarters (77.0%) had practiced for more than 10 years; 25.6% had practiced for more than 20 years. In the 12 months prior to recruitment into the study, physicians treated a mean of 87.7 and 82.3 patients with moderate-to-severe UC and CD, respectively. The mean number of patients treated by gastroenterologists in the past 12 months was greater than those treated by GPs both for UC (96.9 vs 57.4 patients) and CD (91.8 vs 50.9 patients, respectively).

### Physician Treatment Preferences: UC

**Figure 1.**
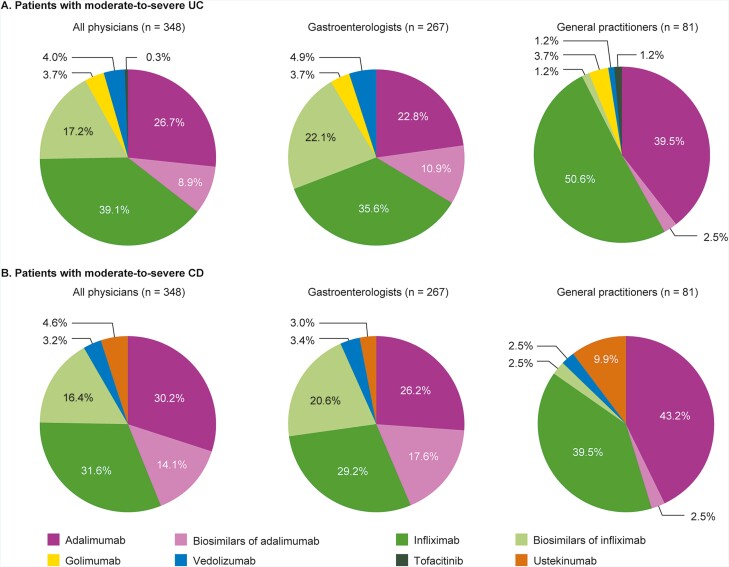
First-line treatment preference of physicians for patients with moderate-to-severe disease, overall and stratified by physician specialty. A, UC; B, CD. Physicians could select only 1 treatment. Abbreviations: CD, Crohn’s disease; UC, ulcerative colitis.

The majority of physicians (95.7%) preferred anti-TNF treatment as first-line biologic for UC, largely infliximab or adalimumab originators (65.8%) or their biosimilars (26.1%), with golimumab indicated as a preferred first-line treatment by only 3.7% of physicians (Figure 1A). There was a marked difference in preference of first-line biologic among physicians. Gastroenterologists were significantly more likely to select biosimilars as their preferred first-line treatment option compared with GPs (33.0% vs 3.7%; *P* < .05). Conversely, GPs had a significantly greater preference for infliximab and adalimumab originators as first-line biologic compared with gastroenterologists (90.1% vs 58.4%; *P* < .05).

The most cited reason for first-line treatment preference was effectiveness (92.8%; Table 1). Improvements in health-related quality of life (67.5% of physicians) and symptoms (51.7%), and decreases in frequency of stools (63.8%) and the presence of blood in stools (50.3%), were cited by more than half of the physicians as the effectiveness-related reasons for their preferences. Availability for the provider (eg, availability on the formulary) and affordability for the patient were more frequently cited as the reasons for preference by physicians who preferred biosimilars as a first-line treatment (42.9% and 30.8% of physicians, respectively), than by physicians who preferred originators (both 11.3% of physicians). Conversely, familiarity was a more frequent reason for preference among physicians who preferred originators than among those who preferred biosimilars (53.7% vs 44.0% of physicians, respectively).

**Table 1.** Reasons selected by physicians for first-line biologic treatment preference in patients with moderate-to-severe UC.

Reasons for treatment preference	All physicians (*N* = 348)	Physician specialty		First-line treatment preference	
		Gastroenterologists (*n* = 267)	GPs (*n* = 81)	Originator biologic (*n* = 257)	Biosimilar (*n* = 91)
Effectiveness	323 (92.8)	248 (92.9)	75 (92.6)	243 (94.6)	80 (87.9)
Treatment improves patient’s quality of life	235 (67.5)	178 (66.7)	57 (70.4)	182 (70.8)	53 (58.2)
Treatment decreases the frequency of stools	222 (63.8)	175 (65.5)	47 (58.0)	172 (66.9)	50 (54.9)
Treatment improves symptoms quickly	180 (51.7)	140 (52.4)	40 (49.4)	129 (50.2)	51 (56.0)
Treatment decreases the presence of blood in the stool	175 (50.3)	139 (52.1)	36 (44.4)	132 (51.4)	43 (47.3)
Treatment reduces diarrhea	171 (49.1)	136 (50.9)	35 (43.2)	124 (48.2)	47 (51.6)
Treatment reduces pain	157 (45.1)	114 (42.7)	43 (53.1)	121 (47.1)	36 (39.6)
Treatment aids in the healing of fistulas	80 (23.0)	69 (25.8)	11 (13.6)	53 (20.6)	27 (29.7)
Treatment reduces incontinence	47 (13.5)	38 (14.2)	9 (11.1)	36 (14.0)	11 (12.1)
Treatment reduces vomiting	52 (14.9)	42 (15.7)	10 (12.3)	43 (16.7)	9 (9.9)
Administrative/patient preference	257 (73.9)	201 (75.3)	56 (69.1)	182 (70.8)	75 (82.4)
Physician familiarity with biologic	178 (51.1)	139 (52.1)	39 (48.1)	138 (53.7)	40 (44.0)
Treatment is available for provider	68 (19.5)	52 (19.5)	16 (19.8)	29 (11.3)	39 (42.9)
Patient preference	58 (16.7)	47 (17.6)	11 (13.6)	47 (18.3)	11 (12.1)
Treatment is more affordable for patient	57 (16.4)	45 (16.9)	12 (14.8)	29 (11.3)	28 (30.8)
Good tolerability	249 (71.6)	188 (70.4)	61 (75.3)	186 (72.4)	63 (69.2)
Other	17 (4.9)	16 (6.0)	1 (1.2)	10 (3.9)	7 (7.7)

Values are presented as *n* (%). Physicians could select multiple reasons for treatment preference. Abbreviations: GP, general practitioner; UC, ulcerative colitis.

**Figure 2.**
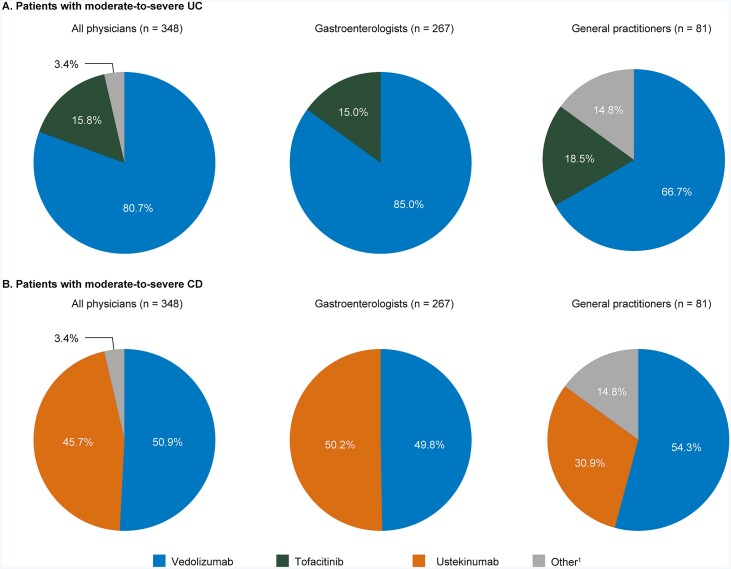
Second-line treatment preference selected by physicians for patients with moderate-to-severe disease in whom anti-TNF treatment had failed, overall and stratified by physician specialty. A, UC; B, CD. Physicians could select only 1 treatment. Anti-TNF treatment included adalimumab, biosimilars of adalimumab, infliximab, biosimilars of infliximab and golimumab. ^1^Other included the physician not prescribing another biologic, the physician referring the patient to another physician and infliximab. Abbreviations: CD, Crohn’s disease; TNF, tumor necrosis factor; UC, ulcerative colitis.

For second-line treatment of patients in whom anti-TNF treatment failed, gastroenterologists predominantly preferred vedolizumab (85.0%), with the remaining 15.0% preferring tofacitinib (Figure 2A). GPs, however, prescribed a more diverse set of second-line therapies, with 66.7% preferring vedolizumab, 18.5% preferring tofacitinib and the remaining 14.8% preferring other treatments (which included the anti-TNF treatment infliximab and the physician not prescribing another biologic and referring the patient to another physician).

### Physician Treatment Preferences: CD

Physician preferences for CD were broadly similar to those reported for UC. Infliximab (31.6% of physicians) and adalimumab (30.2%) were the most preferred first-line therapies, followed by their biosimilars (16.4% and 14.1%, respectively; Figure 1B). Gastroenterologists indicated a significantly greater preference for biosimilars (38.2%) than GPs (5.0%; *P*< .001), although originators were selected more often as first-line treatment options in both groups (55.4% of gastroenterologists; 82.7% of GPs). GP preference for adalimumab originators as first-line treatment was significantly greater than for gastroenterologists (*P* = .004). Approximately 10% of GPs preferred ustekinumab in the first-line setting, compared with only 3.0% of gastroenterologists (*P* = .016).

Effectiveness was the main reason for physician preference for first-line treatment (92.0%). Specific effectiveness-related reasons cited by more than 50% of physicians included improvements in health-related quality of life (64.7%) and symptoms (53.7%), and reductions in frequency of stools (62.1%), diarrhea (52.9%), and pain (53.4%; Table 2). Familiarity with biologic and patient preference were more frequently cited as a reason for choice of first-line treatment by physicians who preferred originators (54.5% and 22.3%, respectively) compared with those who preferred biosimilars (39.6% and 17.0%, respectively). Common reasons for preference included availability for the provider and affordability for the patient, and were cited more frequently as the reasons for preference by physicians who preferred biosimilars as first-line therapy (37.7% and 30.2%, respectively) than by physicians who preferred originators (14.9% and 10.7%, respectively).

**Table 2.** Reasons selected by physicians for first-line biologic treatment preference in patients with moderate-to-severe CD.

Reasons for treatment preference	All physicians (*N* = 348)	Physician specialty		First-line treatment preference	
		Gastroenterologists (*n* = 267)	GPs (*n* = 81)	Originator biologic (*n* = 242)	Biosimilar (*n* = 106)
Effectiveness	320 (92.0)	246 (92.1)	74 (91.4)	226 (93.4)	94 (88.7)
Treatment improves patient’s quality of life	225 (64.7)	174 (65.2)	51 (63.0)	161 (66.5)	64 (60.4)
Treatment decreases the frequency of stools	216 (62.1)	173 (64.8)	43 (53.1)	160 (66.1)	56 (52.8)
Treatment improves symptoms quickly	187 (53.7)	149 (55.8)	38 (46.9)	124 (51.2)	63 (59.4)
Treatment decreases the presence of blood in the stool	163 (46.8)	125 (46.8)	38 (46.9)	121 (50.0)	42 (39.6)
Treatment reduces diarrhea	184 (52.9)	145 (54.3)	39 (48.1)	126 (52.1)	58 (54.7)
Treatment reduces pain	186 (53.4)	136 (50.9)	50 (61.7)	138 (57.0)	48 (45.3)
Treatment aids in the healing of fistulas	76 (21.8)	64 (24.0)	12 (14.8)	46 (19.0)	30 (28.3)
Treatment reduces incontinence	55 (15.8)	44 (16.5)	11 (13.6)	44 (18.2)	11 (10.4)
Treatment reduces vomiting	62 (17.8)	51 (19.1)	11 (13.6)	50 (20.7)	12 (11.3)
Administrative/patient preference	249 (71.6)	192 (71.9)	57 (70.4)	166 (68.6)	83 (78.3)
Physician familiarity with biologic	174 (50.0)	126 (47.2)	48 (59.3)	132 (54.5)	42 (39.6)
Treatment is available for provider	76 (21.8)	57 (21.3)	19 (23.5)	36 (14.9)	40 (37.7)
Patient preference	72 (20.7)	58 (21.7)	14 (17.3)	54 (22.3)	18 (17.0)
Treatment is more affordable for patient	58 (16.7)	48 (18.0)	10 (12.3)	26 (10.7)	32 (30.2)
Good tolerability	230 (66.1)	181 (67.8)	49 (60.5)	161 (66.5)	69 (65.1)
Other	20 (5.7)	19 (7.1)	1 (1.2)	12 (5.0)	8 (7.5)

Values are presented as *n* (%). Physicians could select multiple reasons for treatment preference. Abbreviations: CD, Crohn’s disease; GP, general practitioner.

Gastroenterologist preference for second-line treatment (following failure on anti-TNF treatment) consisted only of vedolizumab and ustekinumab (split approximately 50:50), while for GPs, 54.3% preferred vedolizumab, 30.9% preferred ustekinumab, and 14.8% preferred other treatments (Figure 2B).

### Patient Characteristics

Patient data were based on 1004 patient charts, collected primarily from gastroenterologists (81.8% of charts reviewed). In total, 579 patients with a diagnosis of UC only (France, *n* = 162; Germany, *n* = 272; United Kingdom, *n* = 145), 411 patients with a diagnosis of CD only (France, *n* = 151; Germany, *n* = 162; United Kingdom, *n* = 98), and 14 patients with a diagnosis of both UC and CD (and were included as either patients with UC [*n* = 8] or CD [*n* = 6] according to the indication of their first treatment; these patients were from France [*n* = 1], Germany [*n* = 5], and the United Kingdom [*n* = 8]) (Table 3). In the UC and CD groups, respectively, the mean age was 38.7 and 37.0 years, and 63.4% and 54.6% were male. For patients with UC, the Mayo Endoscopic score was used to classify UC as moderate-to-severe in 73.4% of patients, with clinical impression (60.4%) and other methods (29.5%) also being used. The CDAI was the most commonly used tool for diagnosing CD as moderate-to-severe (63.1% of patients), with clinical impression being used in 52.2% and the HBI being used in 20.9% of patients.

**Table 3.** Demographics and disease characteristics of patients diagnosed with and treated for moderate-to-severe UC or CD.

Characteristic	UC (*n* = 593^a^)	CD (*n* = 425^a^)
Age, years, mean ± SD	38.7 ± 12.9	37.0 ± 12.7
Male	376 (63.4)	232 (54.6)
Type of IBD diagnosis		
UC or CD only	579 (97.6)	411 (96.7)
Both UC and CD	14 (2.4)	14 (3.3)
IBD-related comorbidities^b^	322 (54.3)	238 (56.0)
Anemia	149 (25.1)	127 (29.9)
Anxiety	118 (19.9)	85 (20.0)
Arthritis (nonpsoriatic, nonrheumatoid)	10 (1.7)	10 (2.4)
Asthma	26 (4.4)	28 (6.6)
Cardiovascular disease	10 (1.7)	7 (1.6)
COPD	9 (1.5)	7 (1.6)
Depression	66 (11.1)	35 (8.2)
Diabetes mellitus	17 (2.9)	10 (2.4)
Hypertension	51 (8.6)	22 (5.2)
Joint pain	54 (9.1)	48 (11.3)
Primary sclerosing cholangitis	8 (1.3)	3 (0.7)
Other	8 (1.3)	8 (1.9)
None reported	271 (45.7)	187 (44.0)
Assessment used to classify disease as moderate-to-severe^c^	Mayo Endoscopic score: 435 (73.4)	CDAI: 268 (63.1)
	Clinical impression^d^: 358 (60.4)	HBI: 89 (20.9)
	Other: 175 (29.5)	Clinical impression^d^: 222 (52.2)
		Other: 141 (33.2)
Assessment score used to classify disease as moderate-to-severe: mean ± SD, *n* (%)^c^	Full Mayo score: 8.0 ± 1.5; 420 (70.8)	CDAI: 437.6 ± 153.9; 268 (63.1)
	Endoscopic subscore: 2.5 ± 0.6; 280 (47.2)	HBI: 23.5 ± 21.0; 89 (20.9)
	Rectal bleeding subscore: 2.0 ± 0.7; 259 (43.7)	
	Stool frequency subscore: 2.4 ± 0.7; 260 (43.8)	

Values are *n* (%) unless otherwise stated. Abbreviations: CD, Crohn’s disease; CDAI, Crohn’s Disease Activity Index; COPD, chronic obstructive pulmonary disease; HBI, Harvey–Bradshaw Index; IBD, inflammatory bowel disease; UC, ulcerative colitis.

A total of 14 patients were diagnosed with both UC and CD; 6 patients were excluded from the UC analysis as they were treated for their CD diagnosis, and 8 patients were excluded from the CD analysis as they were treated for their UC diagnosis.

During the 6 months prior to diagnosis.

Physicians could use multiple assessments to classify patients with moderate-to-severe UC or CD. Full Mayo Endoscopic Scores range from 0 to 12, with moderate-to-severe UC classified as a Full Mayo Endoscopic Score from 6 to 12. CDAI scores range from 0 to 600 and HBI scores range from 0 to 43, with moderate-to-severe CD classified as CDAI from 220 to 600 or HBI from 6 to 43.

Physicians were provided examples such as “symptoms” and “number of stools” for the option of classification by clinical impression. Common open text responses for clinical impression included: diarrhea, bloody stools, number of stools, abdominal pain, low hemoglobin levels, weight loss, fatigue, rectal pain, loss of appetite, fever, and tenesmus.

The data relating to treatment during the baseline period were analyzed (UC, *n* = 587; CD, *n* = 417). In this period, 8 patients (1.4%) with UC underwent surgery, with the most common procedures being colectomy with ileo-rectal anastomosis (*n* = 4) and colectomy with ileostomy (*n* = 2). For CD, 47 patients (11.3%) underwent surgery during the same period, with the majority undergoing surgery for abscesses/fistulas (*n* = 17), ileocecal resections (*n* = 15), and fistula removals (*n* = 8). In total, 258 patients with UC (44.0%) and 181 patients with CD (43.4%) received nonbiologic treatments before biologic treatment initiation, with the most common (≥10% of patients) nonbiologic agents in patients with UC being mesalazine (22.3%; *n* = 131) and prednisolone (15.3%; *n* = 90), and in patients with CD being azathioprine (16.3%; *n* = 68) and prednisolone (14.6%; *n* = 61).

The median (interquartile range) length of the postindex observation period was 17.0 (9.9–29.4) months for patients with UC and 21.4 (10.4–38.2) months for the CD cohort.

### Patient Treatment Patterns: UC

**Figure 3.**
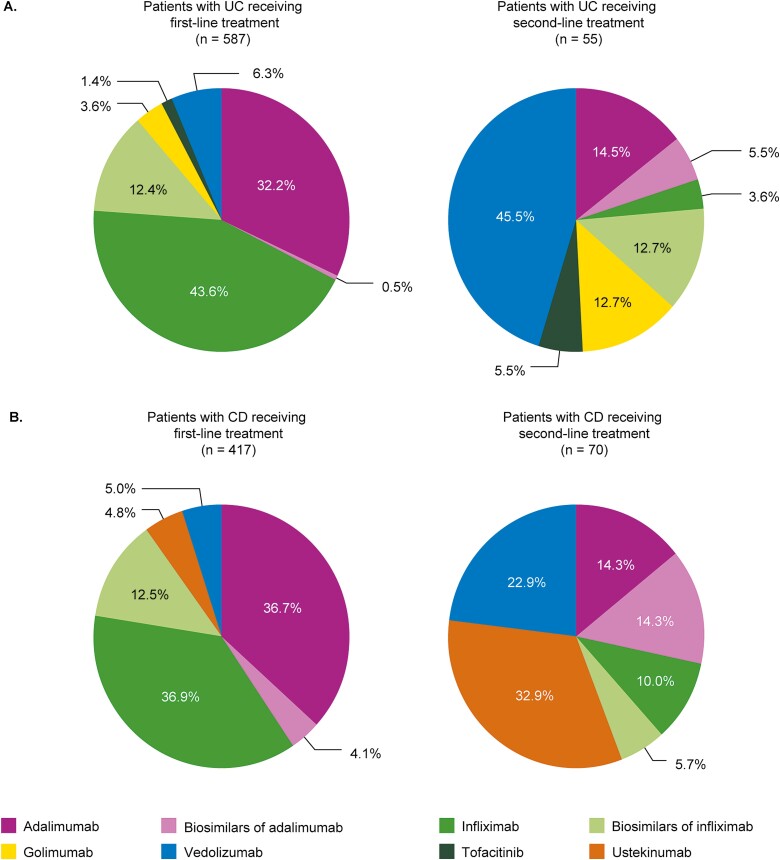
First- and second-line biologic treatments received by patients with moderate-to-severe disease. A, UC; B, CD. Abbreviations: CD, Crohn’s disease; UC, ulcerative colitis.

Figure 3A shows that 92.3% of patients with UC received anti-TNF treatment as their first-line biologic. The originators infliximab and adalimumab were more commonly prescribed to patients as first-line treatments than was the case for their biosimilars (infliximab, 43.6% vs 12.4% of patients; adalimumab, 32.2% vs 0.5% of patients). Persistence (mean ± SD) was generally longer with infliximab (26.6 ± 35.4 months; *n* = 256) and adalimumab (21.2 ± 19.5 months; *n* = 189) than their biosimilars (16.6 ± 9.7 [*n* = 62] to 24.8 ± 20.5 [*n* = 11] months with infliximab biosimilars; and 8.6 ± 1.9 [*n* = 2] to 12.6 ± not calculable [*n* = 1] months with adalimumab biosimilars) (Table 4). As a second-line biologic, 45.5% of patients received vedolizumab, with 49.0% receiving an alternative anti-TNF treatment to that received at first line. Persistence with vedolizumab in the second line was 17.1 ± 15.3 months (*n* = 25). Persistence with second-line biologics was considerably longer for originators than for their respective biosimilars (Table 4).

**Table 4.** Mean persistence with biologic and JAK inhibitor treatment.

Treatment	UC		CD	
	First line (*n* = 587)	Second line (*n* = 55)	First line (*n* = 417)	Second line (*n* = 70)
Infliximab	26.6 ± 35.4; *n* = 256	57.7 ± 0.8; *n* = 2	31.2 ± 29.5; *n* = 154	28.5 ± 30.8; *n* = 7
Infliximab biosimilars				
CT-P13	16.6 ± 9.7; *n* = 62	12.5 ± 3.3; *n* = 5	20.3 ± 12.4; *n* = 47	10.9 ± 11.2; *n* = 2
SB2	24.8 ± 20.5; *n* = 11	5.1 ± 0.8; *n* = 2	10.8 ± 4.1; *n* = 5	29.3 ± 29.2; *n* = 2
Adalimumab	21.2 ± 19.5; *n* = 189	64.6 ± 87.4; *n* = 8	26.7 ± 30.4; *n* = 153	27.2 ± 34.5; *n* = 10
Adalimumab biosimilars				
ABP 501	8.6 ± 1.9; *n* = 2	0; *n* = 0	22.0 ± 39.7; *n* = 11	10.1 ± 8.6; *n* = 7
SB5	12.6 ± NC; *n* = 1	0; *n* = 0	15.3 ± 13.7; *n* = 5	9.4 ± 4.9; *n* = 3
GP2017	0; *n* = 0	3.6 ± 2.2; *n* = 2	8.4 ± NC; *n* = 1	0; *n* = 0
FKB327	0; *n* = 0	8.0 ± NC; *n* = 1	0; *n* = 0	0; *n* = 0
Golimumab	16.3 ± 12.1; *n* = 21	19.3 ± 18.6; *n* = 7	—	—
Tofacitinib	13.4 ± 11.2; *n* = 8	4.0 ± NC; *n* = 3	—	—
Vedolizumab	20.2 ± 14.0; *n* = 37	17.1 ± 15.3; *n* = 25	18.9 ± 12.3; *n* = 21	20.2 ± 15.4; *n* = 16
Ustekinumab	—	—	12.8 ± 9.0; *n* = 20	12.1 ± 8.0; *n* = 23

Values are time in months, mean ± SD. Abbreviations: CD, Crohn’s disease; JAK, Janus kinase; NC, not calculable; UC, ulcerative colitis.

**Figure 4.**
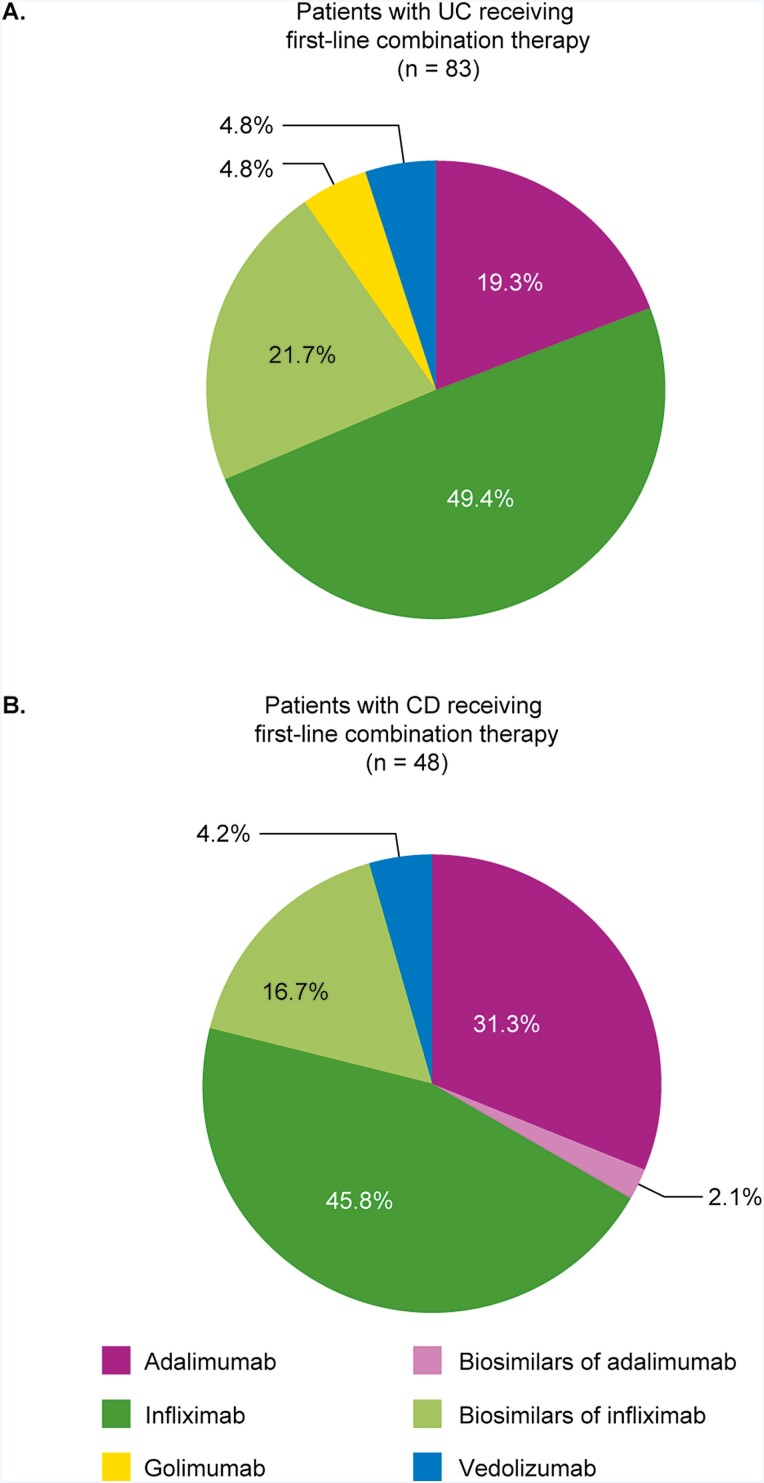
Biologics used as first-line combination treatment with nonbiologic treatments in patients with moderate-to-severe disease. A, UC; B, CD. Abbreviations: CD, Crohn’s disease; UC, ulcerative colitis.

Overall, 83 patients with UC who were treated with first-line biologics (14.1%) received nonbiologic treatment in combination with their biologic treatment. Nonbiologic combination treatment was more frequently prescribed for patients with UC receiving a biosimilar than for those receiving an originator biologic as their first-line treatment (23.7% vs 12.7%, respectively). Nonbiologic treatments were most commonly prescribed in combination with infliximab or biosimilars of infliximab (49.4% and 21.7%, respectively; Figure 4A). Azathioprine was the only nonbiologic treatment used in combination with an originator biologic in more than 5% of patients receiving biologic treatment in the first line and was predominantly used in combination with infliximab (Table 5). In total, 7% (*n* = 18) of patients received azathioprine in combination with first-line infliximab; an additional 1.6% (*n* = 3) and 2.7% (*n* = 1) of patients received azathioprine in combination with first-line adalimumab and first-line vedolizumab, respectively. Azathioprine was also used in patients receiving first-line infliximab biosimilars (CT-P13, 16.1% [*n* = 10]; SB2, 9.1% [*n* = 1]); no patients receiving adalimumab biosimilars received azathioprine.

**Table 5.** Nonbiologic and biologic treatment combinations in first-line therapy in patients with moderate-to-severe UC or CD.

Drug received in the first line	Azathioprine	Methotrexate	Mesalazine	Prednisolone	Other^a^	Total receiving combination
UC (*n* = 587)						83
Infliximab (*n* = 256)	18 (7.0)	5 (2.0)	4 (1.6)	1 (0.4)	13 (5.1)	41
Infliximab biosimilars						
CT-P13 (*n* = 62)	10 (16.1)	0	2 (3.2)	0	5 (8.1)	17
SB2 (*n* = 11)	1 (9.1)	0	0	0	0	1
Adalimumab (*n* = 189)	3 (1.6)	0	5 (2.6)	3 (1.6)	5 (2.6)	16
Adalimumab biosimilars (*n* = 3)	0	0	0	0	0	0
Golimumab (*n* = 21)	0	1 (4.8)	1 (4.8)	1 (4.8)	1 (4.8)	4
Vedolizumab (*n* = 37)	1 (2.7)	1 (2.7)	1 (2.7)	0	1 (2.7)	4
CD (*n* = 417)						48
Infliximab (*n* = 154)	8 (5.2)	5 (3.2)	1 (0.6)	2 (1.3)	6 (3.9)	22
Infliximab biosimilars						
CT-P13 (*n* = 47)	3 (6.4)	0	0	0	4 (8.5)	7
SB2 (*n* = 5)	1 (20.0)	0	0	0	0	1
Adalimumab (*n* = 153)	4 (2.6)	1 (0.7)	0	1 (0.7)	9 (5.9)	15
Adalimumab biosimilars						
SB5 (*n* = 5)	1 (20.0)	0	0	0	0	1
Vedolizumab (*n* = 21)	0	1 (4.8)	0	0	1 (4.8)	2

Values are *n* (%); proportions based on number of patients who received the biologic treatment. Combinations defined as any treatment administered on the same day as or after biologic treatment initiation. Abbreviations: CD, Crohn’s disease; UC, ulcerative colitis.

Other treatments included beclometasone dipropionate, budesonide, ciprofloxacin, cyclosporine, hydrocortisone, mercaptopurine, methylprednisolone, metronidazole, olsalazine, prednisone, sulfasalazine, or any combination of any of the drugs listed in this table.

**Figure 5.**
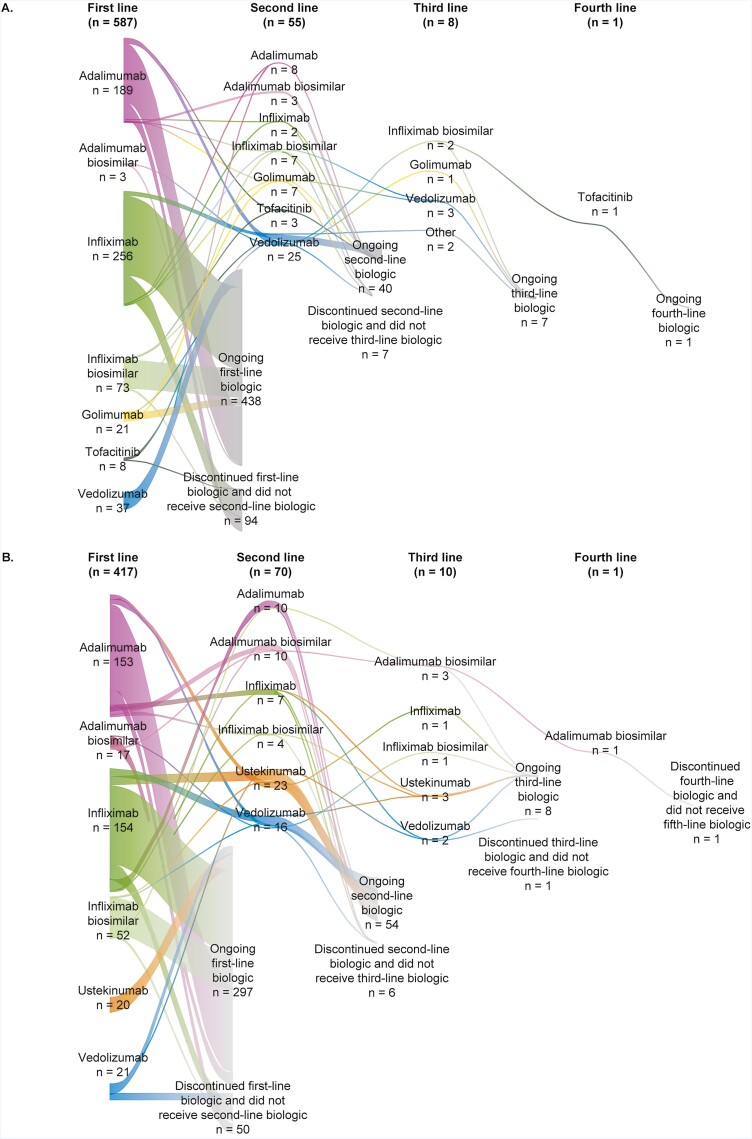
Sankey diagram of biologic treatments for patients with moderate-to-severe disease. A, Ulcerative colitis; B, Crohn’s disease.

For the 587 patients who were treated with biologics for their UC, the biologic treatment sequence is shown in Figure 5A. Most patients (*n* = 532; 90.6%) received only 1 line of biologic treatment during the study; of those, 438 (82.3%) continued to receive that first-line biologic over the study period, with the remaining 94 patients (17.7%) discontinuing and not receiving another biologic. Of the rest of these patients, 55 (9.4%) received a second-line biologic treatment, with 8 (1.4%) and 1 (0.2%) receiving a third- and fourth-line biologic, respectively. In the 250 patients with sufficient follow-up time to be eligible for switching (defined as those who initiated biologic treatment in or before 2017), 42 (16.8%) switched biologic treatment. This difference in rate of switching was even greater when comparing rates of switching among patients who initiated treatment in 2014 or earlier (38.6%) with those initiating in 2019 (2.7%). The most common reason for switching was lack of effectiveness (76.2% of patients); other reasons for switching were: patient/provider preference (11.9%) and cost to the provider (7.1%) and the patient (2.4%).

Overall, 23 patients (3.9%) underwent surgery during the observation period. Colectomy with ileo-rectal anastomosis was the most frequent surgical procedure (47.8%; *n* = 11).

### Patient Treatment Patterns: CD

Of those patients receiving first-line biologic treatment for the treatment of CD (*n* = 417), the originators infliximab (36.9%) and adalimumab (36.7%) were more commonly prescribed than their respective biosimilars (12.5% and 4.1% overall, respectively) (Figure 3B). Persistence (mean ± SD) with first-line biologic treatment was 31.2 ± 29.5 months with infliximab (*n* = 154) and 10.8 ± 4.1 to 20.3 ± 12.4 months with its biosimilars (*n* = 52), with a similar difference in persistence being reported for adalimumab and its biosimilars (26.7 ± 30.4 months [*n* = 153] and 8.4 ± not calculable to 22.0 ± 39.7 months [*n* = 17; the lower value based on a single patient], respectively; Table 4). Of those patients receiving second-line biologic treatment (*n* = 70), more than half received either ustekinumab (32.9%) or vedolizumab (22.9%), with the remaining 44.3% of patients receiving infliximab, adalimumab, or their biosimilars. Persistence with ustekinumab (*n* = 23) and vedolizumab (*n* = 16) was 12.1 ± 8.0 and 20.2 ± 15.4 months, respectively, and persistence with infliximab (28.5 ± 30.8 months; *n* = 7), adalimumab (27.2 ± 34.5 months; *n* = 10), and their biosimilars (10.9 ± 11.2 to 29.3 ± 29.2 months with infliximab biosimilars [*n* = 4]; and 9.4 ± 4.9 to 10.1 ± 8.6 months with adalimumab biosimilars [*n* = 10]) was comparable to when these biologics were used as first-line treatment.

In total, 48 patients with CD (11.5%) received nonbiologics in combination with first-line biologic treatment; the proportion was similar for those receiving first-line originator (*n* = 39/348, 11.2%) or biosimilar (*n* = 9/69, 13.0%) in combination. In these patients (*n* = 48), nonbiologic treatments were most commonly prescribed in combination with infliximab (45.8%) and adalimumab (31.3%) (Figure 4B). There was little consensus on nonbiologic combination use (Table 5), with only azathioprine being used in combination with an originator biologic by more than 5% of patients receiving first-line biologic treatment (in combination with infliximab in 5.2% [*n* = 8]).

For the 417 patients treated with biologics for their CD, biologic treatment is presented in Figure 5B. Overall, 83.2% of patients only received 1 (first-line) biologic treatment, with the majority of these (85.6%) continuing their first biologic while the remaining patients discontinued their first biologic and did not receive a second. In total, 70 patients (16.8%) received second-line treatment, with only 2.4% and 0.2% receiving third- and fourth-line biologic treatment, respectively. Of the 216 patients with sufficient follow-up time to be eligible for switching analysis, 57 (26.4%) switched treatment; the rate of switching was higher for patients initiating treatment in 2014 or before (54.0%) compared with those who initiated in 2019 (3.8%). Lack of effectiveness (66.7%) and patient/provider preference (22.8%) were the most common reasons given for switching, with only 8.8% and 1.8% of switches occurring because treatment was considered too costly for the provider or patient, respectively.

During the observation period, 52 patients with CD (12.5%) underwent surgery. The most frequent surgical procedures were for abscesses/fistulas (34.6%; *n* = 18), fistula removal (28.8%; *n* = 15), and ileocecal resection (19.2%; *n* = 10).

## Discussion

Findings from this real-world evidence study provide an insight into treatment preferences and actual treatment practices among physicians in Europe prescribing biologic therapies for the management of patients with moderate-to-severe IBD. Data suggest that reported treatment preferences are largely in line with current guideline recommendations, while actual treatment practice tends to favor the use of the better-established anti-TNF agents (infliximab and adalimumab) over more recently approved treatments such as golimumab, vedolizumab, ustekinumab, and tofacitinib, possibly reflecting greater availability of or familiarity with the established therapies. Furthermore, anti-TNF originators were used more than their biosimilars, and nonbiologic therapies used in combination with biologic agents were an important feature in the treatment pathways of patients with IBD, although no clear pattern of use was evident in this study.

### Choice of First-Line Biologic Therapy

Despite the recent advent of biosimilars in Europe, our results suggest that physicians in France, Germany, and the United Kingdom have a preference for originators as first-line biologic treatment for both UC and CD that was approximately 2-fold greater than preference for the biosimilars. Effectiveness was the primary reason for treatment preference, with more than 93% of physicians selecting effectiveness as a reason for originator biologic preference in the first line. Conversely, preferences for biosimilars related more strongly to availability and affordability. This result is not surprising given that, in Europe, biosimilars have been reported to be approximately 30% lower in price (on average) than their reference products, according to market research conducted by IMS Health,^11^ and our results are consistent with previous studies suggesting that originators continue to be considered standard of care and that effectiveness is prioritized in the selection of treatments.^7,9,10^

In the current study, a preference for infliximab over adalimumab was seen for UC (56% vs 36%) but not CD (44% vs 48%), and a preference for infliximab for both disorders was observed in actual treatment practice (UC, 56% vs 33%; CD, 49% vs 41%). These results are in agreement with data from the 2017 Adelphi Inflammatory Bowel Disease-Specific Programme, which assessed treatment patterns in 1602 patients with UC in 5 European countries—France, Germany, Italy, Spain, and the United Kingdom—and the United States.^12^ Armuzzi et al reported that in the European countries studied, adalimumab (38%) and infliximab (52%) were the most frequently used biologics in the first-line biologic setting, with similar findings reported in the second line (infliximab 53%; adalimumab 29%).^12^ Similar results were seen in a US study of patients with newly diagnosed UC or CD in the Truven Health MarketScan database (2008–2015), in which 95% of patients in each cohort (UC or CD) received either infliximab or adalimumab as first-line treatment.^13^

In our study, there was a strong preference for originators adalimumab and infliximab over their biosimilars in the therapies prescribed (eg, 44% vs 12% for infliximab originator vs biosimilar in UC), but the difference was less marked in physician preference (39% vs 17%), especially among gastroenterologists (36% vs 22%). This may reflect a change in practice over time; preference data related to clinical practice in 2018, whereas the patient record data relate to the period 2014–2018. Consistent with physician preference for infliximab and adalimumab versus their biosimilars, persistence with an originator was generally longer than with biosimilars in both UC and CD. However, the shorter period of availability for biosimilars compared with originators is likely to contribute to these lower rates by reducing the time over which persistence could be assessed.

### Use of Nonbiologics Added to Biologics

Our study found that a nonbiologic treatment was used in combination with first-line biologic treatment in approximately 10%–15% of patients. The addition of the nonbiologic azathioprine was particularly used in patients with UC receiving infliximab and was used in a higher proportion of patients receiving infliximab biosimilars compared with the originator (9.1%–16.1% vs 7.0%), suggesting that physicians might be more concerned about the potential immunogenicity of infliximab biosimilars compared with the originator. In patients with CD, azathioprine was also used in a higher proportion of patients receiving infliximab compared with other biologics but was not necessarily used more in patients receiving infliximab biosimilars compared with originator, although the patient numbers are too low for meaningful comparisons. These data are broadly in agreement with those reported by Armuzzi et al, who observed that 16% of patients with UC received an immunosuppressant in addition to their first-line biologic, and combination use increased with line of treatment in the EU5 from 16.0% in the first line to 29.1% in the fourth line.^12^ Chen et al also commented on the use of combination treatment (biologic plus immunomodulator) in the United States, finding that combination treatment decreased the risk of nonpersistence (UC and CD combined), but stated that this strategy was more effective when the immunomodulator was started more than 30 days before biologic initiation.^13^

### Choice of Second-Line Therapy and Switching

Physicians in our study showed a strong preference for vedolizumab as the treatment of choice in patients with UC in whom anti-TNF therapy failed, and for patients with CD, vedolizumab and ustekinumab were similarly favored as second-line therapies after anti-TNF failure. However, in clinical practice, switching was uncommon in our study, with approximately 90% of patients with UC and 80% of patients with CD receiving only 1 line of biologic treatment during the observation period. The low rates of switching may in part reflect the limited duration of the observation period because most patients included in the analysis initiated therapy in 2018 or later (median duration of follow-up was 17.0 and 21.4 months for the UC and CD cohorts, respectively). This is supported by the markedly higher rates of switching reported in patients initiating treatment before 2015 compared with in 2019 (UC: 38.6% vs 2.7%; CD: 54.0% vs 3.8%). However, the long duration of time from initiation to discontinuation of first-line biologics observed in our study argues against the low rates of switching merely reflecting the limited follow-up time. Given that data relating to the outcomes of treatment were not collected, it is unclear if the low rate of switching and treatment persistence reflects treatment effectiveness.

The rates of switching observed in our study were lower than have been reported in other studies in Europe and the United States. In a Danish nationwide registry study of incident patients treated with biologics from 2003 to 2016, 23.2% of patients with UC and 34.6% of patients with CD who initiated biologic therapy switched to a second biologic, with approximately 90% of these making the switch within 1 year of stopping the first treatment.^14^ In a Turkish claims database study of patients receiving originator infliximab or biosimilar for rheumatoid arthritis or IBD, the rate of switching from infliximab to a second-line biologic was 14%, which was significantly lower than in patients switching after receiving the biosimilar CT-P13 (51%; *P* < .001; average duration of follow-up was 12 months).^15^ Additionally, in a US claims database study of patients with newly diagnosed UC or CD, rates of switching from infliximab or adalimumab in the first year of therapy were approximately 20%.^13^

### Limitations

The study has several limitations. First, there was a potential selection bias resulting from physicians self-selecting into the study. Second, there was potential bias in the reporting of patient data because these were reported by physicians; however, this was minimized by implementation of quality control and data validation processes. Furthermore, bias resulting from selecting patient charts for inclusion was minimized by employing a computer-generated randomization scheme. Third, limitations may also arise from the complexities of abstracting relevant data using current medical chart standards, which have largely been designed for treating patients and managing patient care. Real-world evidence from medical charts is inherently limited by the availability of clinical data in the medical chart, even though quality assurance procedures and data checks serve to maximize data integrity, and this level of recording may vary across countries and affect comparisons by country, potentially limiting their generalizability beyond France, Germany, and the United Kingdom. Fourthly, the limited duration of the observation period for a substantial proportion of included patients limits the interpretation of evidence relating to second-line therapy and treatment persistence. This is particularly the case for persistence with biosimilars, given the more recent introduction of these drugs. Finally, the data relating to the use of nonbiologics in combination with first-line biologics are limited by the small patient numbers in some treatment groups.

## Conclusions

Treatment patterns in the first-line biologic setting for IBD in France, Germany, and the United Kingdom during the time period of this study are generally in good agreement with current guidelines and with physician preferences. Our data also suggest that many patients persisted with first-line biologic therapies for more than 2 years. Overall, originator biologics continue to dominate the treatment landscape in Europe for treating patients with moderate-to-severe IBD, in terms of both physician preferences and observed treatment patterns. These real-world data provide a valuable reflection of current treatment patterns that can be used to assess the uptake of new treatments as they become available and suggest opportunities for improving treatment options and determining their optimal use among patients with moderate-to-severe IBD. With the growing armamentarium for IBD, further studies are warranted to assess treatment outcomes and establish whether patients are receiving the optimal therapy for their disease.

## Data Availability

No additional data are available.
